# The Clinical Results of Percutaneous Drilling in the Treatment of Chronic Lateral Epicondylitis

**DOI:** 10.7759/cureus.64345

**Published:** 2024-07-11

**Authors:** Sefa Erdem Karapinar, Recep Dincer, Tolga Atay, Yakup Barbaros Baykal, Vecihi Kirdemir, Metin Lutfi Baydar

**Affiliations:** 1 Orthopedics and Traumatology, Suleyman Demirel University, Isparta, TUR

**Keywords:** inflammation, elbow pain, stem cell, percutaneous drilling, lateral epicondylitis

## Abstract

Aim: Lateral epicondylitis is one of the leading orthopedic problems encountered in daily practice. Treatments are more symptomatic than curative. Percutaneous drilling is a minimally invasive method that provides satisfactory results. The aim of this study was to evaluate patients who had undergone percutaneous drilling for chronic lateral epicondylitis.

Material and method: The study included 31 patients who underwent surgical percutaneous drilling because of chronic lateral epicondylitis between 2018 and 2021. The patients were evaluated with respect to demographic characteristics, including age, gender, body mass index (BMI), occupation, education level, hobbies, dominant side, and smoking status. The VAS (Visual Analog Scale) pain scores, PRTEE score (Patient-Rated Tennis Elbow Evaluation - a lateral epicondylitis function scale), and Roles-Maudsly score were examined preoperatively and at one and 12 months postoperatively together with grip strength measured with a Jamar hand dynamometer.

Results: Statistically significant improvements were determined in the VAS score during activity from 8.9 preoperatively to 2.06 at 12 months postoperatively (p<0.01), and in the PRTEE score, from 64.12 preoperatively to 20.61 at 12 months postoperatively (p<0.01). The Roles-Maudsly score at 12 months postoperatively was determined to be excellent in 13 (41.9%) patients, and good in 14 (45.2%). Mean grip strength increased from 69.55 before treatment to 90.97 at the end of 12 months postoperatively.

Conclusion: Autobiological treatments are at the forefront of current treatments for tendinopathies. Percutaneous drilling is a closed method and can be considered an ideal method in the treatment of tendinosis caused by inflammation and mesenchymal stem cells (MSCs) contained in hematoma. It is also an advantageous treatment method for patients with aesthetic concerns as it does not leave any scar tissue and has a low risk of complications.

## Introduction

Lateral epicondylitis, which is also known as tennis elbow, is characterized by pain in the outer section of the elbow. In some patients, pain has been reported to extend to the forearm or toward the shoulder together with pain in the third and fourth fingers. The main area of pain is in a 2.5 cm area at the attachment site of the extensor tendons or in the distal section [1]. Generally, the pain is burning in character and can be triggered by moving the wrist or elbow into extension, making a rotation movement of the wrist, or when gripping a heavy object. Although the pain is usually alleviated with rest, pain that awakens the patient at night or does not recover with rest has been reported in advanced cases. The structure most often damaged is the extensor carpi radialis brevis (ECRB) tendon. A chronic aseptic inflammatory status of this tendon is caused by repeated microtrauma and is the most common cause of musculoskeletal pain in the elbow [2].

Although the pathology has not been fully understood, clinical and physical examinations are more important than imaging in establishing the diagnosis. Despite the common name of tennis elbow, only approximately 10% of patients with lateral epicondylitis play tennis. It is seen at similar rates in males and females and in 1-3% of the general population [3]. The risk of lateral epicondylitis is increased when repetitive movements are performed for at least half an hour on three days or more a week. 

Carrying a load of more than 20 kg and carrying loads of more than 1 kg at least 10 times also increase the risk. There is also known to be an increased risk of lateral epicondylitis in individuals who are obese or who smoke because of the negative effects on vascularity. Different treatment methods have been defined, including rest, orthosis use, ESWT, drugs, exercise, injections, and surgical treatments. Conservative treatments are generally applied but cases of chronic, persistent lateral epicondylitis require a surgical procedure [4].

The aim of this study was to demonstrate that percutaneous drilling is a low-cost procedure with few complications that provides functional and anatomic improvement in patients diagnosed with chronic lateral epicondylitis when conservative treatment has been unsuccessful.

## Materials and methods

Approval for the study was granted by the Ethics Committee of Süleyman Demirel University Medical Faculty (decision no: 355, dated: 23.12.2021). All the patients included in the study provided written informed consent.

The data of these patients followed up in our clinic were collected prospectively. The study was conducted in a single center between 01.01.2018 and 30.12.2021, and all the surgical procedures were performed by a single surgeon.

The study included patients aged 18-70 years with complaints of elbow pain ongoing for at least one year who had undergone conservative treatment, and data were recorded from their files. From a total of 36 patients with chronic lateral epicondylitis, 31 who met the study criteria were included in the analyses. The five patients excluded were because of cervical discopathy in three cases and a history of elbow surgery in two. As there were bilateral complaints in four patients, a total of 35 elbows of 31 patients were evaluated.

A record was made for each patient of age, gender, body mass index (BMI), dominant side, education level, occupation, smoking status, and duration of complaints. Pain scores according to a Visual Analog Scale (VAS), grip strength values, and the PRTEE score (Patient-Rated Tennis Elbow Evaluation - a lateral epicondylitis function scale) were recorded preoperatively and at one and 12 months postoperatively. The Roles-Maudsly score was recorded at one and 12 months postoperatively.

Grip strength measurements with the elbow in flexion and extension were taken using a Jamar hydraulic hand dynamometer before and after the surgical procedure. The measurements were performed with the patient standing and sitting upright, and thus the objective grip strength values of the patients were recorded. Three consecutive measurements were taken with the elbow in 90° flexion and the forearm in a neutral position, with the wrist in mild (0-30°) dorsiflexion, and in mild (0-15°) ulnar deviation. An interval of 30 seconds was waited between each measurement. The average of the three measurements was recorded in pounds/force units for this parameter.

The PRTEE is used to evaluate the pain and functional status of patients with lateral epicondylitis within the last week. The scale has a total of 15 questions with five related to pain and 10 related to functional status. Each item is scored from 10 points, with one point being the best score and 10 being the worst. A total of 50 points can be obtained for pain and the points for functional status are divided by two to provide a total maximum of 50 points, and thus a maximum of 100 points for both pain and function [5].

The Roles-Maudsly score measures patient satisfaction following a surgical procedure and thus evaluates whether or not the patient benefitted from the treatment. Scoring is applied from one to four points, with four points indicating an excellent outcome [6].

Surgical procedure

**Figure 1:**
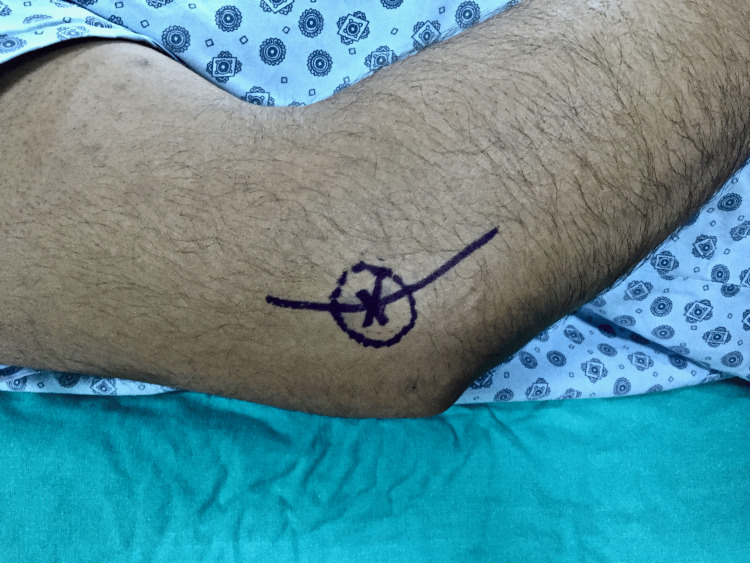
The area was identified and marked under fluoroscopy

All the patients were administered prophylactic antibiotherapy of 1 g cefazolin IV 30 mins before the operation. In all the cases, sedo-analgesia was sufficient for the procedure that was performed under sterile conditions. Fentanyl of 1 mcg/kg, midazolam of 0.05 mg/kg, and propofol of 1 mg/kg were administered at doses appropriate to each patient. Before the procedure, the region of pain on palpation was marked before the anesthetic was administered. The area was sterilized with povidone-iodine and draped appropriately. The area was identified and marked under fluoroscopy (Figure 1).

**Figure 2:**
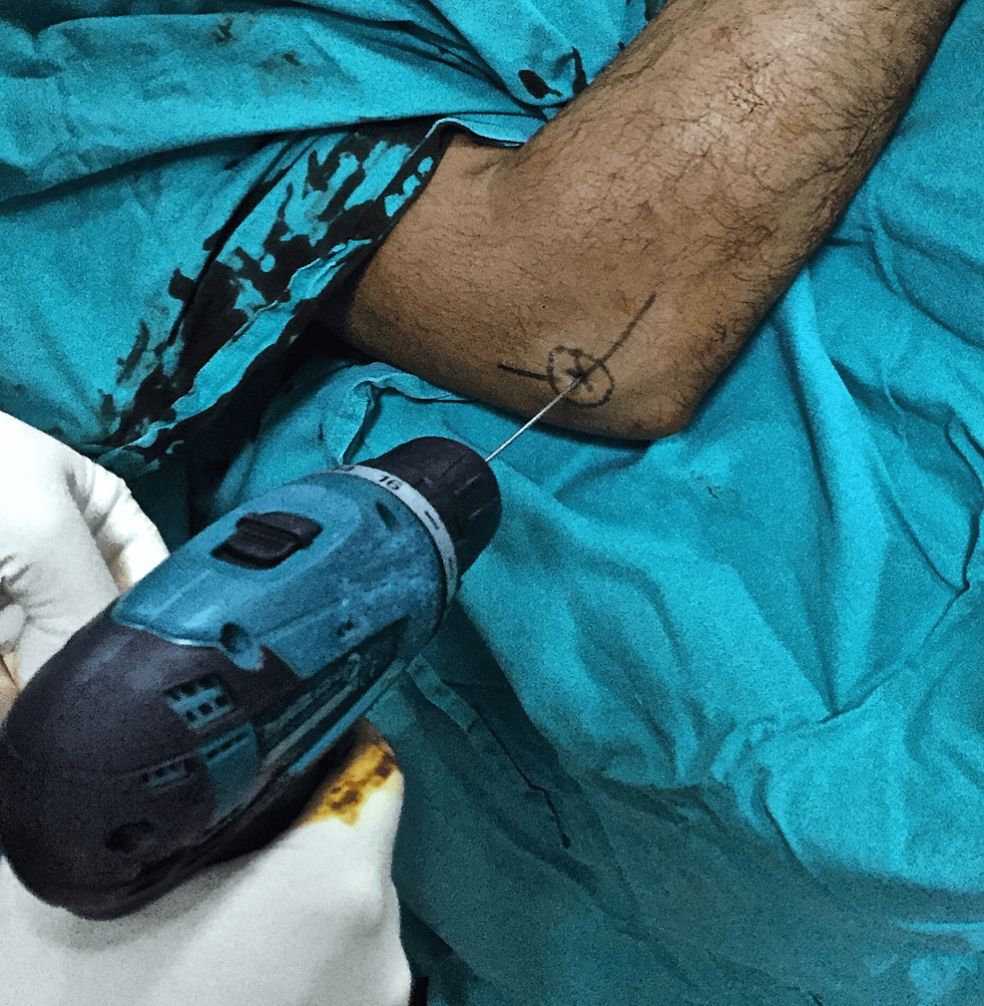
Intraoperative Kirschner wire application

 A 1.6 mm Kirschner wire was advanced to the section approximately 50% of the spongious bone (Figure 2).

From a single skin entry hole, four holes were opened in the bone. Postoperatively, all the patients were applied with a lateral epicondylitis exercise program. 

Statistical analysis

Data obtained in the study were analyzed statistically using SPSS v22.0 software. Descriptive statistics were stated as mean±standard deviation (SD) values for continuous variables and as number (n) and percentage (%) for categorical variables. The Chi-square test was used in the analysis of categorical variables. In the analysis of continuous variables, the non-parametric Mann-Whitney U-test was applied to two independent groups of data not showing normal distribution, and for three or more independent groups, the Kruskal-Wallis test was used. Spearman correlation analysis was applied in the evaluation of relationships between continuous variables. In all the tests, a value of p<0.05 was accepted as statistically significant.

## Results

An evaluation was made of 31 patients, comprising 18 (58.1%) males and 13 (41.9%) females, with a mean age of 43.74±8.76 years (range, 22-63 years). The BMI values showed that 12 (38.7%) patients were of normal weight, 15 (48.4%) were overweight, and four (12.9%) were obese. The education level of the patients was primary school in one (3.2%), high school in 22 (71.0%), and university in eight (25.8%) patients. Occupations were recorded as a housewife for eight (25.8%) patients, manual worker for 13 (41.9%), clerical worker for seven (22.6%), and three (9.7%) were retired. The operation side was right-side in 15 (48.4%) patients, left-side in 12 (38.7%), and bilateral in four (12.9%) (Table 1). The dominant side was right-side in 26 (83.9%) patients and left-side in five (16.1%). Of the total 31 patients, 14 (45.2%) were smokers. 

**Table 1:** Demographic characteristics of the patients

Age (years) (Mean±SD)	43.74±8.76	
BMI median	25.70	
	N	Ratio (%)
Gender		
Male	18	58.1
Female	13	41.9
Operation side		
Bilateral	4	12.9
Right	15	48.4
Left	12	38.7
Dominant side		
Right	26	83.9
Left	5	16.1
BMI		
Normal	12	38.7
Overweight	15	48.4
Obese	4	12.9
Education level		
Primary school	1	3.2
High school	22	71.0
University	8	25.8
Occupational groups		
Housewife	8	25.8
Manual worker	13	41.9
Clerical worker	7	22.6
Retired	3	9.7
Smoking		
Yes	14	45.2
No	17	54.8

The grip strength values measured with a Jamar hand dynamometer were recorded as mean values in pound/force units before treatment and at one month and 12 months after treatment.

The grip strength measured with the elbow in flexion was determined to be 72.87±14.95 preoperatively, 85.23±17.99 at one month postoperatively, and 94.26±22.05 at 12 months postoperatively (Table 2). The increase was statistically significant (p<0.05).

**Table 2:** Grip strength values with the elbow in flexion and extension *Statistically significant

	Mean±SD	p
Grip strength (elbow in flexion)		
Preoperative	72.87±14.95	<0.05*
Postoperative 1st month	85.23±17.99	<0.05*
Postoperative 12th month	94.26±22.05	<0.05*
Grip Strength (elbow in extension)	Mean±SD	
Preoperative	69.55±15.18	<0.05*
Postoperative 1st month	81.23±18.53	<0.05*
Postoperative 12th month	90.07±22.26	<0.05*

When measured with the elbow in extension, grip strength was determined to have statistically significantly increased from 69.55±15.18 preoperatively to 81.23±18.53 at one month postoperatively, and 90.07±22.26 at 12 months postoperatively (p<0.05). The procedure was accepted as successful with the significant increases seen from preoperative to postoperative. 

The VAS score was examined separately in three different conditions; at rest, when lifting a heavy object, and during activity. The improvements from preoperative to postoperative were statistically significant and thus the procedure was accepted as successful (Table 3). 

**Table 3:** VAS score values *Statistically significant

VAS score (at rest)	Median±SD	P (<0.05)
Preoperative	5.60	p<0.05*
Postoperative 1st month	8.34	p<0.05*
Postoperative 12th month	8.12	p<0.05*
VAS score (when lifting a heavy object)	Mean±SD	
Preoperative	8.90±1.13	p<0.05*
Postoperative 1st month	2.58±2.32	p<0.05*
Postoperative 12th month	2.06±2.27	p<0.05*
VAS score (during activity)	Mean±SD	
Preoperative	8.13±1.56	p<0.05*
Postoperative 1^st^ month	2.29±2.31	p<0.05*
Postoperative 12^th^ month	2.00±2.40	p<0.05*

The PRTEE score was determined to be mean 64.12±14.90 preoperatively, 25.70±14.87 at one month postoperatively, and 20.61±17.45 at 12 months postoperatively. In the comparisons from preoperative to postoperative, the improvements were determined to be statistically significant (p<0.05) and thus the procedure was accepted as successful (Table 4).

**Table 4:** PRTEE score values *Statistically significant

	Mean±SD	p (<0.05)
PRTEE score		
Preoperative	64.12±14.90	p<0.05*
Postoperative 1st month	25.70±14.87	p<0.05*
Postoperative 12th month	20.61±17.45	p<0.05*

In the evaluation of patient satisfaction using the Roles-Maudsly score, one patient was not satisfied with the procedure at both one month and 12 months postoperatively. This patient worked in the pediatric blood sampling unit and was thought to have not been satisfied because of their professional status as a healthcare worker. In two patients evaluated as good at one month postoperatively, there was a decrease to partially good status at 12 months postoperatively. The majority of patients were evaluated as good or excellent at both one and 12 months postoperatively, and the procedure was accepted as successful (Table 5).

**Table 5:** Roles-Maudsly score values

Roles-Maudsly score at 1 month after treatment	N	Ratio (%)
1 (excellent)	19	61.3
2 (good)	10	32.3
3 (acceptable-partially good)	1	3.2
4 (poor)	1	3.2
Roles-Maudsly score at 12 months after treatment	N	Ratio (%)
1 (excellent)	13	41.9
2 (good)	14	45.2
3 (acceptable-partially good)	3	9.7
4 (poor)	1	3.2

## Discussion

Chronic lateral epicondylitis is the most frequently seen pathology of the elbow and one of the most common causes of elbow pain. In cases resistant to conservative treatment, the most prominent alternative is surgical treatment [7,8]. In this study, the outcomes of the minimally invasive method of percutaneous drilling were evaluated. With drilling performed in this way, the first response is the formation of a hematoma of bone marrow origin. The content of this hematoma is rich in mesenchymal stem cells (MSC), growth factors, and thrombocytes. As a result of inflammation and the hematoma formed with drilling, cell and vascular migration for matrix development is stimulated. With controlled inflammation providing additional growth factors, MSCs, and increased blood flow have been found to contribute to supplementing the significant deficient materials to increase the chance of recovery [9].

In this study, a 1.6 mm Kirschner wire was used, entered from a single skin hole, and drilling was performed at four points over the lateral epicondyle. It was aimed to reach 50% of the spongious bone. In a study by AbdelNaby et al., percutaneous tenotomy was performed, a drilling procedure at three to four points with a 2.5 mm Kwire [10]. Mueller et al. reported that with open tenotomy and drilling with a 2 mm K-wire, there was thickness because of the danger of fracture that could form in a small area but patient satisfaction was obtained in 80% of the series of 20 patients [11]. In a prospective, double-blinded study by Khasbaba et al., drilling was not performed on patients applied with the Nirschl technique, and there was reported to be a significant improvement in postoperative pain and grip strength [12]. In the current study, it was thought that after drilling, the controlled inflammation in the area with tendinosis accelerated recovery, and the statistically significant rates of improvement obtained in the preoperative to postoperative comparisons showed that the drilling procedure improved the pain and grip strength of the patients.

Previous studies have shown different rates of lateral epicondylitis according to gender. Although some studies have shown similar rates in both males and females, others have reported a higher rate in females [12]. Stasinopoulos et al. stated that lateral epicondylitis was more painful and lasted longer in females than in males [13]. According to the PRTEE scores evaluated preoperatively and at one and 12 months postoperatively, no significant difference was determined according to gender.

Patrick O. Zingg et al. conducted a retrospective study on 21 patients with lateral epicondylitis, in which debridement and drilling were performed with open surgery. Complications were observed in two of these patients. These were hematoma and limited movement [14]. In our study, however, no complications such as hematoma and limited movement were observed. Therefore, we argue that the risk of complications is lower due to the minor nature of percutaneous surgery compared to open surgery.

Different outcomes of lateral epicondylitis have been reported according to BMI. Werner et al. reported that there was an association between BMI and tendinitis [15]. In the current study, according to the PRTEE scores evaluated preoperatively and at one and 12 months postoperatively, no statistically significant difference was determined according to the BMI groups. The VAS scores also showed no significant difference.

According to the study of Buchanan et al., although a definite relationship between lateral epicondylitis and smoking has not been established, smoking is also among the risk factors. It is shown as a risk factor because it disrupts the nutrition of the tendon [16]. In our study, it was obtained that the mean PRTEE score before treatment, one month after treatment, and 12 months after treatment did not show a statistically significant difference according to smoking status.

Studies comparing open and percutaneous surgical techniques are insufficient. A randomized controlled trial comparing open and percutaneous surgery, originally presented by Nirschl and Pettrone, was published in 2004. The group that underwent percutaneous surgery returned to work and social life more quickly. Although advances in surgical techniques continue, the question of whether the best treatment is open surgery, arthroscopic surgery, or percutaneous surgery is still controversial [17]. Arthroscopic methods are also used in the treatment of lateral epicondylitis. A study by Jerosch and Schunck, which included 20 patients, showed that arthroscopic treatment was also successful. However, the ability to see pathology with arthroscopy is lower than with the open method [17].

Limitations of the study to be considered were that physiopathological healing could not be shown with MRI in the first postoperative year after percutaneous drilling, the number of patients was relatively low, no prospective comparison could be made with other surgical methods (open debridement and arthroscopic procedures), and that there were no long-term follow-up results. Further studies to overcome these limitations would be able to contribute further to the literature.

## Conclusions

Although multiple alternative treatment methods have been presented for this disorder, there is still no established treatment algorithm. Many methods have been tried, from exercise therapy to drug therapy. Surgical procedures have been resorted to as a last resort. Surgical procedures have not been organized according to a definitive protocol among themselves. Orthobiological treatments are at the forefront of current treatments for tendinopathies. Percutaneous drilling is a closed method and can be considered an ideal method in the treatment of tendinosis caused by inflammation and MSCs contained in hematoma. It is also an advantageous treatment method for patients with aesthetic concerns as it does not leave any scar tissue and has a low risk of complications. We think that it is a method that can be applied to patients who have previously received conservative treatment but whose pain has not gone away. Being a closed method has advantages over open surgery. Rehabilitation with a physiotherapist after the surgical procedure will be more efficient in terms of recovery.
